# New Direct Oral Anticoagulants (DOAC) and Their Use Today

**DOI:** 10.3390/dj4010005

**Published:** 2016-03-11

**Authors:** Heike Schwarb, Dimitrios A. Tsakiris

**Affiliations:** Heike Schwarb, Diagnostic Hematology, University Hospital Basel, CH-4031 Basel, Switzerland; heike.schwarb@usb.ch

**Keywords:** oral anticoagulants, non-VKA oral anticoagulants (NOAC), DOAC, rivaroxaban, apixaban, edoxaban, dabigatran

## Abstract

The ideal anticoagulant is oral, has a wide therapeutic range, predictable pharmacokinetics and pharmacodynamics, a rapid onset of action, an available antidote, minimal side effects and minimal interactions with other drugs or food. With the development of the novel direct oral anticoagulants (DOAC), we now have an alternative to the traditional vitamin K antagonists (VKA) for the prevention and treatment of thrombosis. DOACs have limited monitoring requirements and very predictable pharmacokinetic profiles. They were shown to be non-inferior or superior to VKA in the prophylaxis or treatment of thromboembolic events. Particularly in terms of safety they were associated with less major bleeding, including intracranial bleeding, thus providing a superior benefit for the prevention of stroke in patients with atrial fibrillation. Despite these advantages, there are remaining limitations with DOACs: their dependence on renal and hepatic function for clearance and the lack of an approved reversal agent, whereas such antidotes are successively being made available. DOACs do not need regular monitoring to assess the treatment effect but, on the other hand, they interact with other drugs and interfere with functional coagulation assays. From a practical point of view, the properties of oral administration, simple dosing without monitoring, a short half-life allowing for the possibility of uncomplicated switching or bridging, and proven safety overwhelm the disadvantages, making them an attractive option for short- or long-term anticoagulation.

## 1. Background

Population ageing due to stagnation and declining population growth and ever-increasing life expectancy are marks of the demographic development over the last decades in industrialized countries. It is predicted that this aging process will continue up until the mid-21st century. This means that the proportion of elderly people will increase significantly in percentage, which will in turn be reflected in an increase in medically compromised patients.

Cardiovascular disease (including venous thrombotic events) represents the most frequent diagnosis in medical practices/hospitals and continues to be the leading cause of death in statistics.

Furthermore, the landscape of treating these patients has changed in the recent years. Overall, patients with atrial fibrillation (AF) initiated on anticoagulant treatment for stroke prevention increased from 57.4% to 71.1%. Use of vitamin K antagonists (VKA, coumarin derivates) and antiplatelet agents (combined or alone) fell from 83.4% to 50.6%, while the use of novel direct oral anticoagulants (DOAC), with or without antiplatelet agents, increased from 4.1% to 37.0% [1].

This phenomenon extends also to the population needing dental services. The treatment of the elderly with cardiovascular disease and pre-existing medication will occupy a larger space in dental practices. Therefore, issues relating to the properties and use of the new direct oral anticoagulants become more and more important.

**Figure 1:**
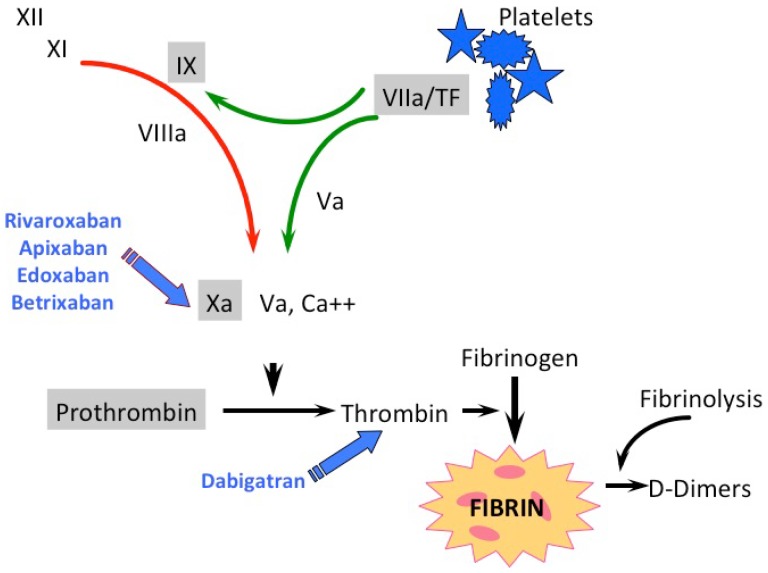
Classical scheme of the coagulation cascade with direct oral anticoagulants (DOAC) and vitamin K antagonists (VKA) attack points. Coagulation enzymes are presented in roman numerals; green arrows depict the extrinsic and the red arrow the intrinsic coagulation pathway; the gray-shaded coagulation factors are vitamin K-dependent.

For many decades heparins and VKAs were the most commonly used anticoagulants. VKAs reduce the synthesis of functional vitamin K-dependent coagulation enzymes (factors II, VII, IX, X, as well as protein C and protein S) by interfering with the vitamin K redox cycle. Although effective, they were characterized by well-known limitations. Aiming to overcome these limitations, new anticoagulants have been developed in recent years, which were specifically directed against an activated clotting factor, either factor II (thrombin, FIIa) or factor Xa (FXa) (Figure 1). They should present fewer drug interactions and a greater therapeutic index than VKAs, while maintaining the same effectiveness.

## 2. Nomenclature of Oral Anticoagulants

Various terms have been used to describe the new class of oral anticoagulants, although they are not so new or novel anymore. Terms in the medical literature include: novel/new oral anticoagulants or non-VKA oral anticoagulants (NOAC), direct oral anticoagulants (DOAC), and target-specific oral anticoagulants (TSOAC). However, the use of multiple terms and abbreviations can lead to confusion among providers and patients. The term NOAC has been used the longest, but there is at least one reported account where the term NOAC written in the medical record was interpreted as meaning “No AntiCoagulation”, potentially resulting in the withholding of a medication that is needed [2]. For this reason, we prefer to use the term DOAC.

## 3. Pharmacokinetic Profiles of DOAC

### 3.1. Direct Oral Factor IIa-Inhibitor

Dabigatran etexilate is the first established factor IIa (thrombin)-inhibitor. It is a prodrug, which is converted into the active form dabigatran by microsomal carboxylesterases in the liver. Due to a low bioavailability of 6%, high doses of dabigatran etexilate are needed to achieve a proper anticoagulatory effect [3].

It is mainly eliminated through the kidneys at a rate of 80%, and dabigatran therefore is not suitable for patients with renal insufficiency (Table 1).

### 3.2. Direct Oral Factor Xa-Inhibitors

Rivaroxaban was the first approved factor Xa inhibitor. FXa is a clotting factor at a crucial turn in the coagulation pathway leading to thrombin generation and clot formation.

Rivaroxaban rapidly, reversibly and highly selectively binds human factor Xa, for which it has a >1000-fold greater selectivity than for other biologically relevant serine proteases. It provides its effectiveness in a concentration-dependent manner.

The mechanism of action of rivaroxaban and all other factor Xa inhibitors is the inhibition of prothrombinase complex-bound and clot-associated factor Xa, resulting in a reduction of the thrombin burst during the propagation phase of the coagulation cascade. They do not directly affect platelet aggregation induced by collagen, adenosine diphosphate or thrombin, but by inhibiting factor Xa, they indirectly decrease clot formation induced by thrombin [4]. It is eliminated in active form by the kidneys to an extent of 33%.

Apixaban is another highly selective and reversible inhibitor of free and clot-bound factor Xa. After oral administration it is absorbed rapidly and reaches steady state plasma concentrations in three days (taken twice daily) with only mild accumulation. Most of the administered dose is is eliminated in the feces, and about 25% is recovered in the urine [5]. The area under the plasma concentration-time curve (AUC) was 32% higher in elderly subjects compared with younger volunteers; only a modest increase was seen in women *versus* men [6].

Edoxaban is a once-daily oral anticoagulant that rapidly and selectively inhibits factor Xa in a concentration-dependent manner. It undergoes biotransformation into various metabolites; the most abundant is formed through hydrolysis. Edoxaban is eliminated in feces and urine, and a lower proportion of the administered dose is eliminated by the kidneys (50%) in comparison to dabigatran (80%), apixaban (27%) and rivaroxaban (33%) [7].

## 4. Indications

The registered indications of all DOACs are almost identical. Dabigatran, Rivaroxaban, Apixaban and Edoxaban are approved for lowering the risk of stroke and embolism in patients with nonvalvular AF (NVAF), deep vein thrombosis (DVT) prophylaxis, treatment and secondary prophylaxis of DVT and pulmonary embolism (PE) in Europe and the USA. With the exception of Edoxaban, they are indicated for the prevention of venous thrombotic events (VTE) in knee or hip replacement surgery patients as well. Rivaroxaban has also recently been approved in Europe only for the secondary prevention of acute coronary syndrome (ACS); rivaroxaban administered with acetylsalicylic acid (ASA), alone or with ASA plus clopidogrel, is indicated for the prevention of atherothrombotic events in adult patients with elevated cardiac biomarkers after ACS. This indication is not registered in the USA.

There has been an effort to extend the indication profile to other clinical entities, such as mechanical heart valves, primary prophylaxis after general surgery or hospitalization in internal medicine wards, but appropriate randomized trials produced inconclusive or negative results concerning efficiency and safety of DOACs in these settings, so these indications have been abandoned.

With the now existing wider range of opportunities in anticoagulation, choosing the best-tailored drug is important. In particular, secondary diagnoses and co-medication are especially to be considered. In the GARFIELD-AF Registry, the largest and longest-running registry of patients with newly diagnosed AF and at least one additional stroke risk factor, the use of anticoagulants was more frequent in patients with moderate to severe chronic kidney disease. Furthermore, one-year outcomes in 17,159 patients with AF reveal differences between patients with moderate to severe chronic kidney disease (n = 1760) and those with no or mild chronic kidney disease (CKD). Moderate to severe chronic kidney disease was associated with a twofold higher rate of mortality and major bleeding and a 1.4-fold higher rate of stroke [1,8]. Therefore, the increased use of anticoagulants in these patients is warranted but also requires an accurate weighing of possible interactions.

## 5. Relevant Drug-Drug Interactions and Criteria for Dose Reduction

The fact that most of the DOACs are substrates of P-glycoprotein induces a potential risk of drug-drug interactions. Relevant interactions are known for antiarrhythmics (Dronedarone, Amiodarone, Digoxin, Chinidin, Propafenon, Verapamil), antihypertensives (Carvedilol, Felodipin, Nifedipin, Timolol, Propranolol, Labetalol, Diltiazem, Aliskiren), antiplatelet drugs (Clopidogrel, Ticagrelor, Dipyridamol), statins (Atorvastatin, Lovastatin), oncologics, antibiotics (Erythromycin, Clarithromycin, Rifampicin, Fluconazol, Ketoconazol), and HIV protease inhibitors (Ritonavir).

### 5.1. Dabigatran

Dabigatran is metabolized by P-glycoprotein. It should be avoided in conjunction with P-glycoprotein inducers (e.g., rifampicin). Furthermore, avoid the coadministration of P-glycoprotein inhibitors (e.g., dronedarone, ketokonazol) when creatinine clearance (CrCl) is <30 mL/min. Dose adjustment is generally required, depending on the degree of renal impairment.

### 5.2. Rivaroxaban

Rivaroxaban is metabolized by CYP3A4 and P-glycoprotein. It should be avoided in conjunction with P-glycoprotein and strong CYP3A4 inhibitos (e.g., ketoconazol, itraconazol, ritonavir). Furthermore, avoid the coadministration of P-gylcoprotein and strong CYP3A4 inducers (e.g., carbamazepin, phenytoin, phenobarbital, rifampicin, St. Johns wort). Dose adjustment is generally required, depending on the degree of renal impairment.

### 5.3. Apixaban

Apixaban is metabolized by CYP3A4 and is substrate of P-glycoprotein. It should be avoided in conjunction with strong dual inducers of CYP3A4 and P-glycoprotein (e.g., rifampicin, phenytoin, phenobarbital). Dose adjustment is generally required, depending on the degree of renal impairment according to the manufacturer’s instructions.

### 5.4. Edoxaban

A very limited proportion of edoxaban (<4%) is metabolized by cytochrome P450. Intestinal transport, as compared to other DOACs, occurs through the P-glycoprotein (P-gp) efflux transporter mechanism. Dose adjustment is generally required, depending on the degree of renal impairment according to the manufacturer’s instructions.

## 6. Is Adherence Still a Problem?

Adherence was evaluated in patients with acute coronary syndrome taking aspirin, betablockers and statins. Only 21% took all three drugs; 71% took aspirin, 46% took betablockers and 44% took statins [9]. So for elderly medically compromised patients who have to take more than one drug, the risk of non-compliance is significantly higher.

The In-Range study, a prospective cohort study of adults initiating warfarin at two anticoagulation clinics, was performed to determine factors affecting non-adherence to warfarin. Interestingly, warfarin was not taken on 21% of patient-days. Risk factors are related to education level, employment status, mental health functioning and cognitive impairment [10]. Therefore, a lack of monitoring may make adherence with the new drugs worse and short half-lives leave patients at risk if adherence is poor.

## 7. Monitoring of DOACs

Normally there is no indication for anticoagulation monitoring for the DOACs, and drug levels should not be followed or used for dose adjustments.

However, the assessment of drug exposure and the anticoagulant effect may be needed in special clinical situations such as for patients who present with renal or hepatic insufficiency, potential drug-drug interactions, suspected overdosing, a need for urgent surgery or emergency situations, such as serious bleeding or thrombotic events [11].

Factor Xa inhibitors can be assayed with anti-factor Xa-based chromogenic assays using specific calibrators; alternatively, a simple anti-FXa assay similar to that used for heparins can be utilized. In the latter case, observed values of therapeutic intensity differ considerably from those of the heparins, so a careful estimation and validation have to be carried out. In its investigator’s brochure, each manufacturer has published observed levels of the respective drug activity for various indications (Table 2).

There are no data available for anti-factor Xa activity on threshold values indicating bleeding or thrombotic risk under treatment with DOACs.

## 8. Interferences, Effect or Lack of Effect on Routine Coagulation Tests

The presence of DOACs in plasma samples interferes with all coagulation assays, which use a coagulation-endpoint, in a concentration-dependent manner [14]. For the interpretation of a coagulation assay in a patient treated with a DOAC, in comparison to VKA, it is even more important to know when the drug was administered in relation to the time of blood sampling. A coagulation assay obtained on a blood sample taken 3 h after intake of the DOAC at peak level will demonstrate a much larger impact on the coagulation test than when performed at the trough concentration, *i.e.*, 12 or 24 h after ingestion of the same dose.

DOACs can prolong various clotting tests and the available coagulation assays do not provide a reliable and accurate prediction of a patient’s coagulation status. In general, the global coagulation assays cannot be used to assess the intensity of anticoagulation with a DOAC.

*Dabigatran:* The coagulation effect can be globally observed using activated partial thromboplastin time (APTT), ecarin clotting time (ECT) and thrombin time (TT). All tests are artificially prolonged in the presence of Dabigatran.

The relationship between dabigatran and the APTT is curvilinear [15]. Conversely, a normal APTT or TT in dabigatran-treated patients has been used in emergency situations to exclude any relevant remaining anticoagulant effect and even to guide decisions on urgent interventions [16].

The ECT assay provides a direct measure of the activity of thrombin inhibitors, but is not readily available in most of the labs. A normal TT excludes the presence of dabigatran in the sample.

*Rivaroxaban:* The coagulation effect can be observed using prothrombin time PT/INR, aPTT and anti-factor Xa activity (=Heparin activity). Among the most widely available tests, the PT is more sensitive than the APTT, but is not specific and the response varies according to the thromboplastin used as a reagent.

*Apixaban:* Apixaban prolongs PT and APTT only at supra-pharmacological concentrations. If necessary, PT may provide qualitative assessment of the presence of apixaban, but normal values do not rule out the presence of apixaban.

*Edoxaban:* Prolongs PT and APTT, but in a variable way. No known relation to a bleeding risk is described.

DOACs also interfere with thrombophilia testing and other functional assays of coagulation factors. The presence of DOACs renders false positive lupus anticoagulant assays, and artificially increased protein C and protein S activity values if a coagulation endpoint assay is used for these tests. Antithrombin activity is also found to be falsely high if factor Xa is used in the assay as a substrate. Therefore, a time window of at least 24 h is recommended between the last intake of a DOAC and blood sampling to confidently assess the coagulation parameters. This time window may be even longer if renal insufficiency is present (≥48 h). Genetic testing for thrombophilia (factor V and factor II polymorphisms) is not affected by the DOAC.

## 9. Managing the Risk of Periprocedural and Spontaneous Bleeding with DOACs

The potential for bleeding must be weighed against the risk of thrombosis before discontinuing any DOAC prior to surgery [17,18,19]. If possible, surgery should be postponed until there is a balance of risk to benefit. For scheduled surgery or invasive procedures that have a low risk of hemorrhagic complications, certain time windows are recommended for safe surgery. Rivaroxaban, apixaban and edoxaban should be stopped 24–36 h and dabigatran 24–72 h before the intervention, depending on the renal function. Anticoagulation should be resumed as soon as it is safe to do so and after adequate hemostasis has been achieved.

When elective surgery is associated with a moderate or high risk of bleeding, guidelines recommend that DOACs be withheld for up to five days prior to the intervention whenever possible [18,19]. In general, there is no need to bridge a DOAC with low molecular weight heparins in the perioperative setting. Comparable pharmacokinetic allows management with DOACs alone.

The timing of discontinuation of dabigatran, which is eliminated primarily through the kidneys, should be based on a patient’s renal function indicated by Creatinin Clearance CrCl [20]. Importantly, the duration of time dabigatran needs to be withheld should be doubled in the setting of moderate renal insufficiency (CrCl < 50 mL/min) [13]. In a recent study, a standardized interruption scheme for dabigatran in patients with elective surgery increased rates of “no-residual dabigatran effect” and reduced bleeding to a minimum [21].

If oral medication cannot be taken after surgical interventions, consider administering a parenteral anticoagulant.

## 10. Management of Unexpected Bleeding

Direct antidotes for all DOACs have been recently produced and successfully tested for efficacy and safety in phase I/II trials (Table 1) [22,23,24], and phase III trials are ongoing. Idarucizumab is a humanized monoclonal antibody Fab-fragment, which binds specifically to dabigatran and reverses the anticoagulant effect immediately. Andexanet alfa is a recombinant FXa decoy, binds directly FXa inhibitors and immediately reverses the anticoagulant effect. Arapazine (PER977) is a third small synthetic molecule, which binds both FXa- and FIIa-inhibitors, thus acting as a “universal” antidote [25]. Currently only the preparation against dabigatran is available for clinical use on a named patient use program (idarucizumab, PraxBind^®^). Both andexanet alfa and arapazine are not yet registered for clinical use.

When bleeding occurs under DOACs and no direct antidote is available, procoagulant measures such as substitution with four factor prothrombin complex (4fPCC, 30–50 IU/Kg BW) or with factor eight bypassing agents (FEIBA^®^ 1 × 50 IU/kg of body weight Bolus) is a first option [26,27]. The initial use of human recombinant activated factor seven has been subsequently abandoned as inefficient. However, the use of 4fPCC, being more broadly used, has lately been the subject of controversy [28]. The use of tranexamic acid or e-aminocaproic acid as anti-fibrinolytics may also add to the hemostatic effect. Hemodialysis is an option only for dabigatran to eliminate the drug. Substitution with 4fPCC (30 IU/kg of body weight Bolus) is a less preferable option for dabigatran.

## 11. How to Switch between Anticoagulants

When transitioning from a VKA to a DOAC, the VKA is discontinued and dabigatran or apixaban can be administered when a patient’s INR is <2.0; rivaroxaban and edoxaban may be started when the INR is <2.5 [29,30,31,32].

When switching from a DOAC to a VKA, the DOAC is withdrawn when INR reaches therapeutic levels. Caution is needed here to avoid the preanalytical effect of the drug on the INR-value. It is recommended that blood samples be taken for INR measurements at the trough level of the drug, *i.e.*, just before the next intake, in order to avoid unnecessary technical interference with the assay. Another valid option seen in national recommendations (USA) is to stop the DOAC and start the VKA, often with no overlap of the two. LMWH bridging is used if it is felt that the patient needs additional protection to maintain therapeutic anticoagulation during the transition.

Replacing intravenous unfractionated heparin (UFH) by a DOAC can be started once intravenous UFH (half-life +2 h) is discontinued. Care should be taken in patients with CKD where the elimination of heparin may take longer.

When changing from low-molecular-weight heparin (LMWH) to a DOAC, the latter can be initiated at the place of the next scheduled LMWH dose.

When switching from a DOAC to a parenteral anticoagulant (UFH or LMWH), this can be initiated at the place of the next scheduled dose of the DOAC [33].

## 12. DOAC in Cancer Patients with Thrombosis

Thromboembolic events are a common complication in cancer patients and are considered a bad prognostic feature for cancer survival. LMWH have been successfully used in this setting and are considered superior to VKAs in terms of efficacy and safety. DOACs as oral agents would be a very attractive alternative. There are only post-hoc subgroup analyses for Rivaroxaban and Dabigatran in patients with tumor thrombosis, showing a similar efficacy and a tendency towards less major bleeding in comparison to VKAs, but the very low patient numbers in the studies prohibit sufficient conclusions [34,35,36]. Moreover, there are no randomized trials of DOACs *versus* LMWHs. Therefore, in the guidelines of most oncology scientific societies, LMWH is still the gold standard for patients with VTE and cancer [37,38].

## 13. Perspectives

With three direct factor Xa inhibitors already approved, covering all the identical spectrum of indications, a subsequent drug needs to carry special advantages in the pharmacodynamics and kinetics. This requirement is met by Betrixaban–another direct FXa inhibitor—by being barely excreted via renal clearance and therefore able to be optimally used in patients with renal insufficiency. Betrixaban is currently not registered for any indication, neither in the United States nor in Europe [39].

Further direct anticoagulants based on the principle of single factor inhibition of coagulation are in development [40]. Factor IXa, factor XIa and factor VIII are further interesting targets in the coagulation cascade. An exemplary case of a direct factor VIIIa-inhibitor is the recombinant human monoclonal antibody (MoAb TB-402) that binds with high affinity to the C1 domain of Factor VIII, partially inhibiting the action of factor VIII. Acting as a partial factor VIII inhibitor with considerably long elimination half-life and allowing a single injection for a one- or two-week coverage, TB-402 is designed to prevent thrombosis while maintaining haemostasis. Phase II studies in patients with orthopedic surgery are already underway. Also noteworthy is the development of a dual thrombin/factor Xa inhibitor like EP217609 or Tanogitran. Whether these agents will reach the level of clinical use still remains to be proved. Interestingly, further development of new agents is frequently interrupted or abandoned, due to the high performance level of the already existing comparators.

## Figures and Tables

**Table 1:** Pharmacodynamics and pharmacokinetics of DOAC (once daily (OD); twice daily (BID); P-glycoprotein (P-gp); area under the curve (AUC); heparin induced thrombocytopenia (HIT); maximum drug concentration in plasma (Cmax)).

DOAC	Rivaroxaban	Edoxaban	Apixaban	Dabigatran
	Xarelto ^®^	Lixiana ^®^	Eliquis ^®^	Pradaxa ^®^
Target	FXa	FXa	FXa	FIIa
t ½	7–13 h	10–14 h	8–15 h	12–17 h
Cmax	2–4 h	2–4 h	2–4 h	1–2 h
Renal clearance	33% active 33% inactive	50%	25%	80%
Bioavailability	80%	62%	50%	6%
Dosing scheme	OD	OD	BID	BID
Interaction	CYP3A4, CYP2J2, P-gp	P-gp	CYP3A4 P-gp	P-gp
Interference with food	Increases AUC to 39%	None	None	Prolongs Cmax to 2 h
Antidote	Andexanet alfa	Andexanet alfa	Andexanet alfa	Idarucizumab
Allowed in pregnancy	No	No	No	No
Induces HIT II	No	No	No	No

**Table 2:** Usual observed levels of DOACs depending on the dosage and the time of sampling [12,13], (once daily (OD), twice daily (BID)).

DOAC	Dosing Schedule	Total Trough (ng/mL) Median (P10-P90)	Total Peak (ng/mL) Median (P10-P90)	Anti-Xa Maximum (IU/mL)	Anti-Xa Minimum (IU/mL); (Median)
Dabigatran	110 mg BID	66 (28–155)	133 (52–275)		
	150 mg BID	93 (40–215)	184 (74–383)		
Rivaroxaban	15 mg BID	57 (20–140)	229 (180–320)		
	20 mg OD	25.6 (5.93–86.9)	255 (189–419)		
Apixaban	2.5 mg BID			1.3 (0.67–2.4)	0.84 (0.37–1.8)
	5 mg BID			2.55 (1.36–4.79)	1.54 (0.61–3.43)
Edoxaban	30 mg OD			2.1	0.35 (0.21–0.57)
	60 mg OD			3.8	0.64 (0.37–1.12)

## Abbreviations

The following abbreviations are used in this manuscript:

ACS	acute coronary syndrome
AF	atrial fibrillation
APTT	activated partial thromboplastin time
ASA	acetylsalicylic acid
AUC	area under the plasma concentration-time curve
BID	twice daily
CKD	chronic kidney disease
CrCl	creatinine clearance
ECT	Ecarin clotting time
FIIa	activated factor II
FXa	activated factor X
DOAC	direct oral anticoagulant
DVT	deep vein thrombosis
HIT	Heparin-induce thrombocytopenia
LMWH	low molecular weight heparin
NVAF	non-valvular atrial fibrillation
OD	once daily
4fPCC	four factor prothrombin complex concentrate
PE	pulmonary embolism
PT	prothrombin time
rFVIIa	recombinant factor VIIa
SEE	systemic embolic event
t ½	half life
TSOAC	target specific oral anticoagulant =DOAC= NOAC
TT	Thrombin time
UFH	unfractionated heparin
VKA	Vitamin K Antagonist
VTE	venous thrombotic event
